# Integrated Transcriptomic and Metabolomic Analyses of Cold-Tolerant and Cold-Sensitive Pepper Species Reveal Key Genes and Essential Metabolic Pathways Involved in Response to Cold Stress

**DOI:** 10.3390/ijms23126683

**Published:** 2022-06-15

**Authors:** Chonglun Gao, Muhammad Ali Mumtaz, Yan Zhou, Zhuang Yang, Huangying Shu, Jie Zhu, Wenlong Bao, Shanhan Cheng, Liyan Yin, Jiaquan Huang, Zhiwei Wang

**Affiliations:** 1 Key Laboratory for Quality Regulation of Tropical Horticultural Crops of Hainan Province, College of Horticulture, Hainan University, Haikou 570228, China; gaochonglun_37@163.com (C.G.); alimaken07@gmail.com (M.A.M.); 13937103464@163.com (Y.Z.); yangzhuang99@163.com (Z.Y.); hnhyshu@163.com (H.S.); jasperjie@aliyun.com (J.Z.); wlbao@hainanu.edu.cn (W.B.); 990865@hainanu.edu.cn (S.C.); 2 Hainan Yazhou Bay Seed Laboratory, Sanya Nanfan Research Institute of Hainan University, Sanya 572025, China; 3 Key Laboratory for Sustainable Utilization of Tropical Bioresources of Hainan Province, College of Tropical Crops, Hainan University, Haikou 570228, China; lyyin@163.com

**Keywords:** transcriptome, metabolomics, molecular mechanism, cold stress, pepper

## Abstract

Cold stress, triggered by particularly low temperatures, is one of the most severe forms of abiotic stress in pepper plants and a major constraint to the global pepper industry, threatening crop production and food security. To acclimatize to extreme conditions, the plant undergoes numerous modifications, including genetic and metabolic modulations. A thorough study of both the genetic and metabolic alterations of plants in response to cold stress is vital to understanding and developing the cold stress resistance mechanism. This study implemented transcriptome and metabolome analyses to evaluate the cold stress response in cold-tolerant and cold-sensitive pepper species. The weighted gene co-expression network revealed three significant modules related to cold stress tolerance in *Capsicum pubescens*. We identified 17 commonly enriched genes among both species at different time points in 10 different comparisons, including the *AP2* transcription factor, LRR receptor-like serine, hypersensitivity-related 4-like protein, and uncharacterized novel.295 and novel.6172 genes. A pathway enrichment analysis indicated that these DEGs were mainly associated with the MAPK signaling pathway, hormone signaling pathway, and primary and secondary metabolism. Additionally, 21 significantly differentially accumulated metabolites (DAMs) were identified in both species after 6 h of cold stress. A transcriptome and metabolome integrated analysis revealed that 54 genes correlated with metabolites enriched in five different pathways. Most genes and metabolites involved in carbohydrate metabolism, the TCA cycle, and flavonoid biosynthesis pathways were upregulated in cold-tolerant plants under cold stress. Together, the results of this study provide a comprehensive gene regulatory and metabolic network in response to cold stress and identified some key genes and metabolic pathways involved in pepper cold tolerance. This study lays a foundation for the functional characterization and development of pepper cultivars with improved cold tolerance.

## 1. Introduction

Plants constantly encounter unfavorable growth conditions in the modern era because of climate change [1]. Maintaining transitional or constant stressful conditions in the external environment causes physiological, biochemical, and molecular modifications in plants, influencing growth, development, and yield responses [2]. It causes chronic or long-term damage to cellular, metabolic, and molecular functions [3]. To acclimatize to extreme conditions, the plant metabolic system undergoes numerous modifications. Reactive oxygen species (ROS) perform rudimentary functions in plant adaptation to abiotic stresses [4]. They act primarily as signal transduction molecules, controlling a variety of pathways involved in plant stress adaptation. However, they are also potentially harmful byproducts of stress metabolism. Antioxidant enzymes such as catalase and peroxidase can limit or scavenge ROS [5]. Additionally, ROS can activate various signaling pathways that regulate the expression of specific receptive genes, including the mitogen-activated kinase (MAPK) signaling pathway [6].

During the cold stress response, the cellular concentrations of various osmoregulatory metabolites (such as glucose, sucrose, and galactose) rise [7]. Certain low-molecular-weight compounds (such as betaine, glycine, and proline) help plants maintain their osmotic resistance, cell turgor, and water consumption and metabolism [8]. Numerous studies have established that the diverse quantitative characteristics of cold tolerance involving various regulatory and metabolizing processes are related to antioxidant enzyme activation, transcription factor (TF) regulation, the expression of cold-responsive genes, hormone signaling transduction, and primary and secondary metabolite concentrations [9,10,11,12]. Changes in the number of sugars, amino acids, and TCA cycle intermediates are the most noticeable metabolic responses to cold stress due to the modulation in gene expression [13,14]. Meanwhile, integrating transcriptomics and metabolomics enables researchers to consider the gene-to-metabolite pathways involved in cold stress.

Capsicum, or chili pepper, is extensively cultivated worldwide as a vegetable or spice. *C. annuum*, *C. assamicum*, *C. baccatum*, *C. chinense*, *C. pubescens*, and *C. frutescens* are the most frequently grown Capsicum species. Pepper contains high concentrations of carotenoids, vitamin C, vitamin E, capsaicinoids, and flavonoids, which are beneficial to human health [15]. Capsaicinoids are extensively used in traditional medicine due to their high medicinal properties. Capsaicin has been used as a pain reliever in various chemotherapies and radiation treatments, and it has been shown to reduce the risk of cancer in some studies [16]. In 2018, the amount of pepper produced worldwide stood at 752,000 tones, jumping by 5.1% from the previous year. In general, the total output indicated a conspicuous expansion from 2007 to 2018 at an average annual rate of +3.2% over the last eleven years (Research and Markets, 2020). The pepper plant is native to the American tropical region and is highly vulnerable to cold temperatures. Pepper plants grow well between 21 °C and 27 °C, and the growth is decelerated below 12 °C and above 30 °C [17]. 

An integrated analysis of the transcriptome and metabolome is an effective way to understand genome function and the related pathways. Numerous studies have been carried out using transcriptome and metabolome analyses for cold stress in numerous crops [18,19]. However, no studies have considered the entire spectrum of transcriptional and metabolic reactions to cold stress in cold-tolerant (*C. pubescens*) and cold-sensitive (*C. chinense*) pepper species. In the present study, we merged transcriptomic and metabolomic studies to elaborate the transcriptional and metabolic modulations in cold-tolerant and cold-sensitive pepper species to discover possible linkages between cold-responding genes and metabolite accumulations. These findings provide an opportunity to understand more about the molecular mechanisms that control cold assimilation in peppers.

## 2. Results

### 2.1. Response of Morphological and Physiological Indexes of Pepper Leaves under Cold Stress

**Figure 1:**
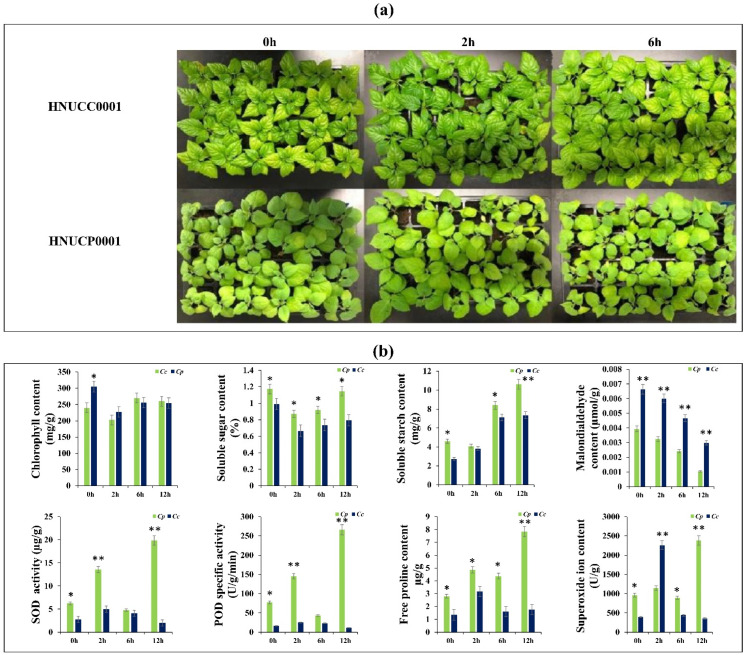
(**a**) Phenotypic representation of the *C. pubescens* and *C. chinense* pepper plants subjected to cold stress at different time points. (**b**) Response of morphological and biochemical indexes of the *C. pubescens* and *C. chinense* pepper plants subjected to cold stress at different time points. Data represent means ± S.E. of three replicates. Asterisks indicate significant differences (* *p* > 0.05, ** *p* > 0.01, Student’s *t*-test).

In our study, there was no apparent morphological difference in the response to cold stress in the first 2 h, but *C. chinense* leaves began to wilt after 6 h and eventually started dying after 12 h of cold stress, whereas *C. pubescens* leaves showed no wilting symptoms (Figure 1a). To further understand the morphological changes in pepper leaves, we used transmission electron microscopy to analyze the ultrathin sections of pepper tissue. There were no significant differences in the number, size, and morphology of the chloroplasts found in the leaves of the cold-treated and the non-treated plants, but the cells seemed to produce more starch grains. Additionally, the chloroplast grana lamellae in the *C. chinense* were looser due to the plant stress response (Figure S1). We measured the chlorophyll content and the soluble sugar and starch contents to determine the effect on plant photosynthetic potential. The total chlorophyll content of *C. chinense* leaves exposed to cold stress decreased significantly compared to the leaves of *C. pubescens* (Figure 1b). The amount of carbohydrates in the leaves of *C. chinense* was also reduced to some extent, but the starch content increased as the stress cycle was prolonged (Figure 1b). MDA is the end result of lipid peroxidation by reactive oxygen species (ROS) in the cell membrane, and its concentration is commonly used to determine the degree of cell damage [20]. The MDA level was significantly higher in *C. chinense* plant leaves than the *C. pubescens* leaves. Cold stress simultaneously increased SOD and POD enzyme activity in *C. pubescens* and *C. chinense* leaves, but the degree of increase was much higher in *C. pubescens* leaves compared to the *C. chinense* leaves. The SOD and POD activities increased linearly for the first 6 h, then dropped precipitously at 12 h but remained significantly higher than the control (Figure 1b). Proline is an advantageous solute for increasing cellular osmolarity in response to abiotic stress. The consistency of free proline was compared in control and cold-treated groups of *C. chinense* and *C. pubescens* plants to elucidate the proline response to cold stress. Proline was detected at various time points following cold stress, with higher levels in 12 h *C. pubescens* plants than in controls, whereas the proline content decreased steadily in cold-treated *C. chinense* leaves (Figure 1b). Compared to the control, the O^•2^ content dramatically increased after 2 h but declined at 6 h and 12 h in *C. chinense* leaves. In contrast, in *C. pubescens* leaves O^•2^ increased steadily and peaked at 12 h of cold treatment (Figure 1b).

### 2.2. RNA Sequencing Analysis under Cold Stress

**Figure 2:**
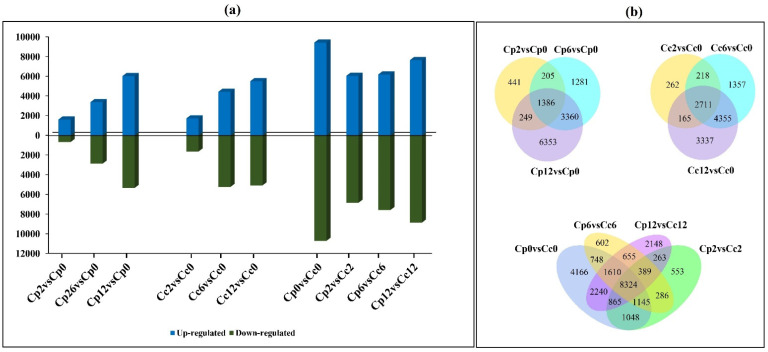
Summary of the RNA-seq results (**a**) The overall number of upregulated and downregulated genes in the cold-treated *C. pubescens* and *C. chinense* pepper seedlings at different time points (**b**) Venn diagram representation of commonly expressed genes in different comparisons of cold-treated *C. pubescens* and *C. chinense* pepper seedlings at different time points.

The expression profile of cold-treated and non-treated pepper plants of *C. pubescens* and *C. chinense* were analyzed using RNA-seq. A total of 6 GB of clean data was obtained for each sample. After screening the raw data, 44–65 M clean reads were obtained with an error rate of 0.03. The percentage of Q20 was more than 95%, the percentage of Q30 was more than 88%, and the content of GC was more than 41% (Table S1). Comparing the obtained clean reads with the reference genome, at least 82% of the clean reads in *C. chinense* were mapped to the reference genome, of which more than 78% were uniquely located; at least 68% of the clean reads in *C. pubescens* were mapped to the reference genome, of which more than 65% were uniquely located. A total of 20145 DEGs were identified in different comparisons among and between both species. Interestingly, the number of DEGs increased in both species as the stress period prolonged (Figure 2a). Furthermore, 1386, 2711, and 8324 commonly enriched DEGs were identified in different comparisons (Figure 2b). A complete list of DEGs is presented in Supplementary File S1.

### 2.3. Weighted Gene Co-Expression Network Analysis under Cold Stress

**Figure 3:**
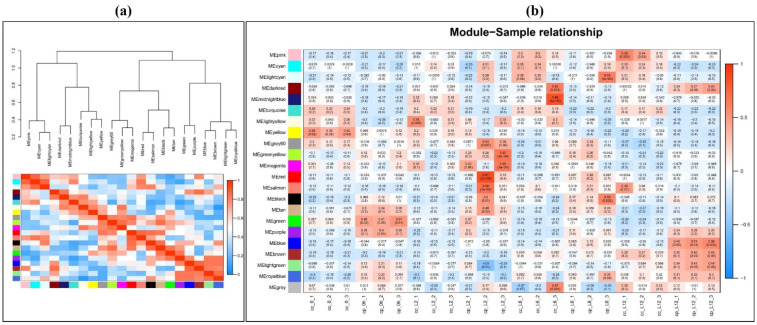
WGCN analysis of cold-tolerant and cold-sensitive samples under different time durations of cold stress. (**a**) Genes were assigned to one of the 22 modules. The sequence diagram is shown at the top of the image. The bottom of the figure shows the different color gene modules and the number of bases they contain. (**b**) Correlation analysis between module and sample. The number on each cell is the correlation coefficient between each module gene and sample, and the number below is the corresponding *p*-value. Among these modules, the blue module is the most relevant to cold resistance.

To identify groups of genes whose expression was directly correlated with cold stress tolerance, a co-expression analysis was performed, and 21 major modules were generated. The co-expressed genes were grouped based on expression profile similarities into color-coded modules, which were further separated by hierarchical clustering. The network heatmap of 1,000 randomly selected genes indicates a relatively high level of independence among these clusters (Figure 3a). Statistical eigengene expression was also found to be significantly correlated with the blue (r = 0.87, *p* < 0.001), black (r = 0.5, *p* < 0.001), and purple (r = 0.51, *p* < 0.01) module genes showing a strong positive correlation with cold stress tolerance (Figure 3b). The genes in these modules were involved in multiple crucial pathways including, the MAPK signaling pathway, starch sucrose metabolism, phenylpropanoid biosynthesis, and plant hormone signaling pathways (Supplementary File S2).

### 2.4. Cold-Stress-Responsive Transcription Factor Prediction

**Figure 4:**
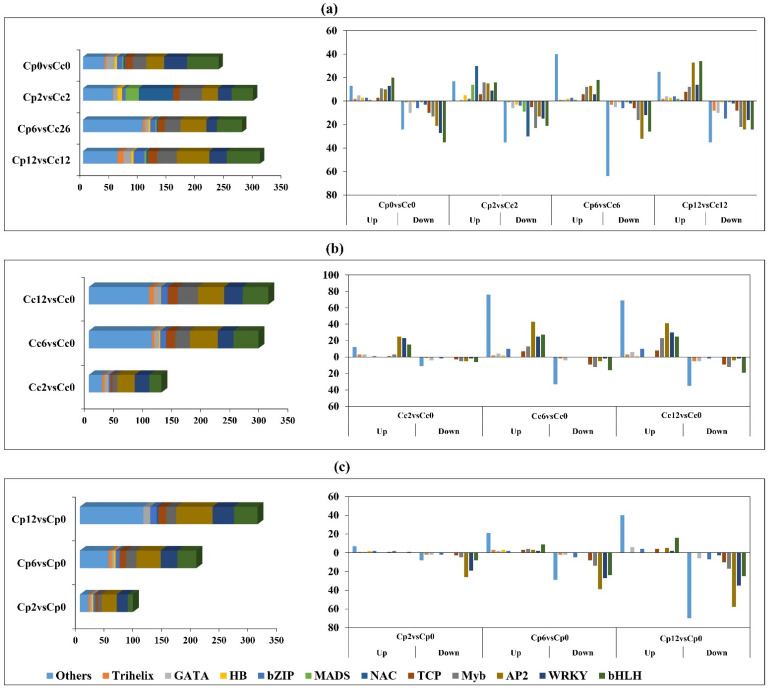
Cold-stress-responsive TF prediction (**a**) Family distribution of the transcription factors and number of upregulated and downregulated transcription factor family members occurring in the *C. pubescens* vs. *C. chinense* at different time points of cold stress. (**b**) Family distribution of the transcription factors and number of upregulated and downregulated transcription factor family members occurring in *C. pubescens* at different time points of cold stress (**c**) Family distribution of the transcription factors and number of upregulated and downregulated transcription factor family members occurring in *C. chinense* at different time points of cold stress.

We found 11 families with at least one gene matching the DEG dataset among the 81 TF families predicted in the *Arabidopsis* TF database. In response to cold stress, 332 transcription factors were identified in *C. pubescens* leaves, including 231 that were upregulated and 101 that were downregulated at 12 h of cold stress (Figure 4a). In most TF families, the number of upregulated genes was higher than the number of downregulated genes during cold stress compared to the control. A total of 326 transcription factors were identified in *C. chinense* leaves, including 152 that were upregulated and 174 that were downregulated at 12 h of cold stress (Figure 4b). In most TF families, the number of genes that were downregulated was higher than the number of genes that were upregulated during cold stress compared to the control. While comparing *C. pubescens* vs. *C. chinense*, there was more upregulation of some TF families, such as *HSF*, *FAR*, and *bHLH*. Additionally, more active participants were identified in the *GRAS*, *WRKY*, *AP2*, *BZIP*, *MYB*, *DOF*, and *ARF* families, all of which play key roles in regulating cold-sensitive structural genes (Figure 4c).

### 2.5. KEGG Pathway Mapping of DEGs

**Figure 5:**
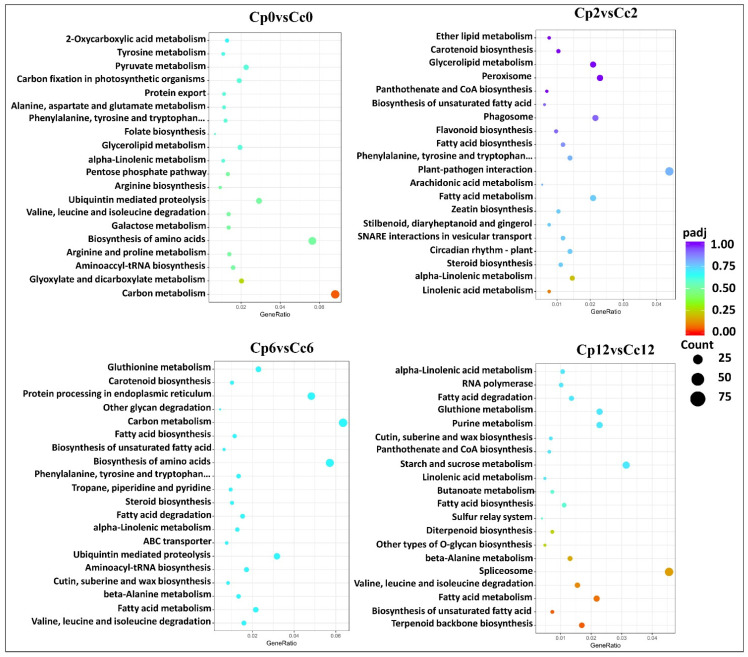
KEGG enrichment analysis of DEGs in the cold-treated *C. pubescens* and *C. chinense* pepper seedlings. The ordinate indicates the pathway name, and the abscissa is the enrichment factor. The size of the circle indicates the number of genes enriched in the KEGG pathway, and the color of the circle represents the *p*-value.

A KEGG annotation-based enrichment analysis of *C. pubescens* vs. *C. chinense* revealed the involvement of DEGs in primary and secondary metabolic pathways. In particular, genes involved in carbohydrate metabolism and amino acid metabolism were enriched, including sucrose metabolism, galactose metabolism, and citrate cycle metabolism (TCA cycle) genes that were enriched in *C. pubescens* leaves in response to cold stress as compared to *C. chinense*. Other secondary metabolism pathways were also significantly enhanced, including flavonoid biosynthesis, phenylalanine metabolism, valine metabolism, riboflavin metabolism, serine, threonine metabolism, glycine, and arginine and proline metabolism (Figure 5, Supplementary File S3). Furthermore, the plant MAPK signaling pathway has been generally proven to be strongly conserved and has a crucial function in plant responses to abiotic stress. Here, the KEGG annotation found that the cold stress impaired the MAPK signal pathway in *C. chinense* leaves compared to *C. pubescens* (Figure 5). The MAPK signaling route may also be active in controlling cold-strength reactions in *C. pubescens*, although only three DEGs were downregulated in cold-stress-treated *C. pubescens* leaves (Supplementary File S3).

### 2.6. Photosynthetic-Pathway-Related Genes in Response to Cold Stress

**Figure 6:**
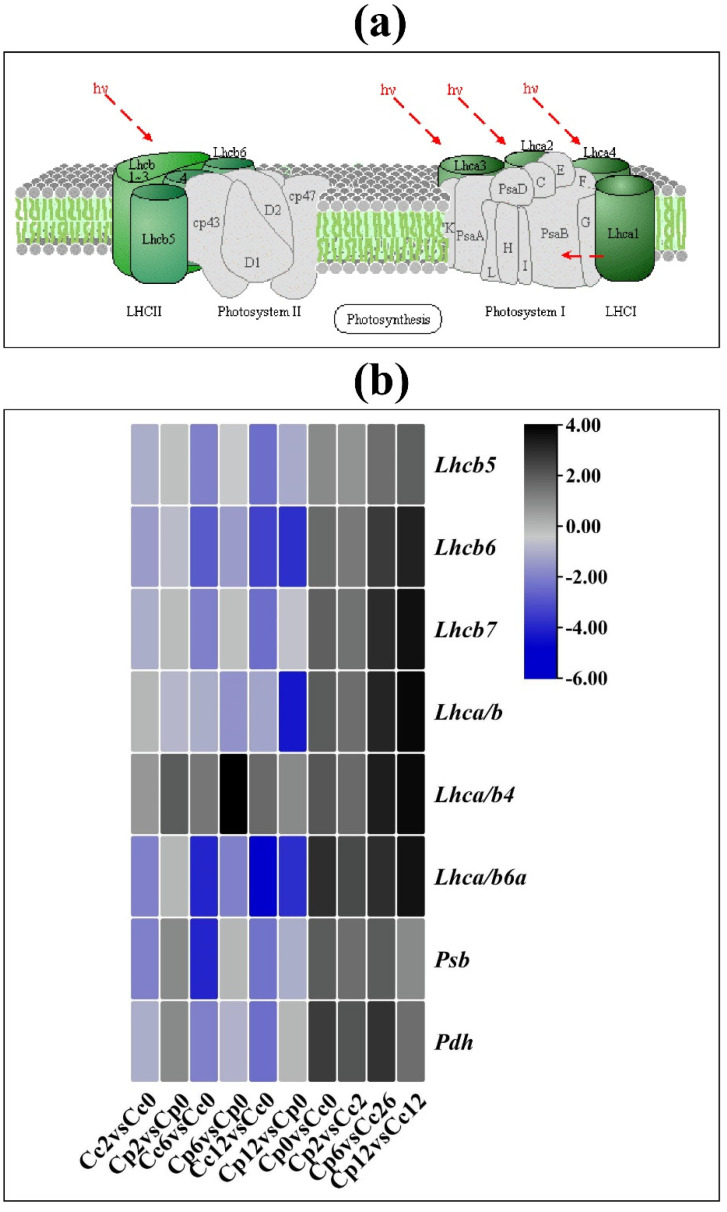
(**a**) Schematic representation of light harvesting pathway in plants (KEGG: Kyoto encyclopedia of genes and genomes). (**b**) Differentially expressed photosynthetic pathway genes in the cold-treated *C. pubescens* and *C. chinense* pepper seedlings at different time points.

In total, eight differentially expressed genes were identified related to photosynthesis antenna protein in both *C. pubescens* and *C. chinense* leaves under control and stress conditions. We observed the downregulation of most genes as the cold stress time prolonged in both species. However, interestingly, while comparing *C. pubescens* vs. *C. chinense*, most of the genes, including *Lhcb6*, *Lhcb7*, *Lhca*/*b*, and *Lhca*/*b4,* showed higher expression patterns, suggesting the photosynthetic pathway was less impaired in *C. pubescens* compared to the *C. chinense* leaves (Figure 6, Supplementary File S1).

### 2.7. Plant Hormone Signal Transduction Pathway in Response to Cold Stress

**Figure 7:**
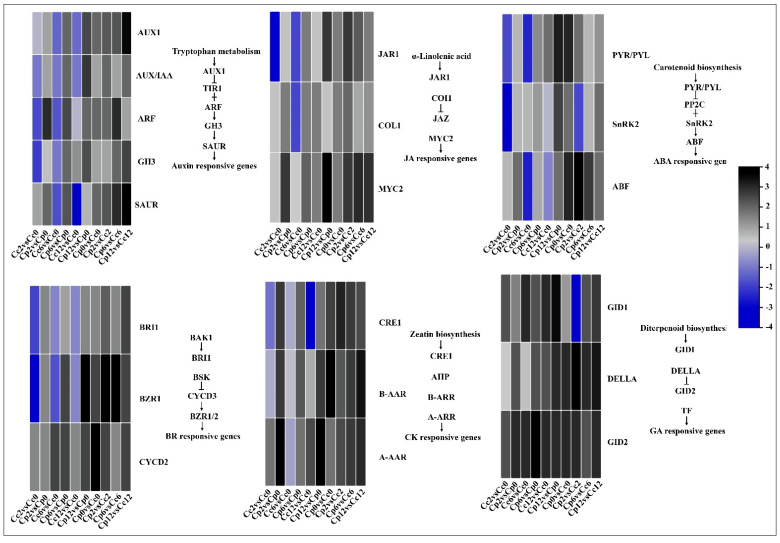
Differentially expressed hormone signaling pathway genes in the cold-treated *C. pubescens* and *C. chinense* pepper seedlings.

Hormone signaling is critical for hormone-induced biochemical changes in plant growth, development, and environmental responsive pathways. In this study, five DEGs involved in auxin signal transduction were enriched. *Aux1*, *GH3*, and *SaUR* were significantly upregulated at 12 h of cold stress in *C. pubescens*. Furthermore, the expression of these genes was significantly expressed in *C. pubescens* compared to *C. chinense*. Cytokinin signal transduction, gibberellin signal transduction, abscisic acid (ABA) signal transduction, ethylene signal transduction, brassinolide signal transduction, salicylic acid (SA) signal transduction, and jasmonic acid (JA) signal transduction were also identified (Figure 7, Supplementary File S1). Among the ABA-related genes, *PYR*/*PYL* and *ABF* were most enriched in *C. pubescens* at 12 h cold stress. Interestingly, there was no significant change in gibberellin-related genes in *C. pubescens* vs. *C. chinense* in non-treated plants, but during the cold stress period, gibberellin-related genes showed higher expression levels in *C. pubescens* plants compared to *C. chinense.* The change in gibberellin-related genes in response to cold stress suggests their involvement in the cold-stress tolerance of *C. pubescens* seedlings. Interestingly the *BZR1*/*2* transcription factor responsible for inducing BR-regulated genes showed significantly higher expression in *C. pubescens* leaves under stress conditions than *C. chinense* (Figure 7).

### 2.8. Metabolic Profiling Analysis in Response to Cold Stress

**Figure 8:**
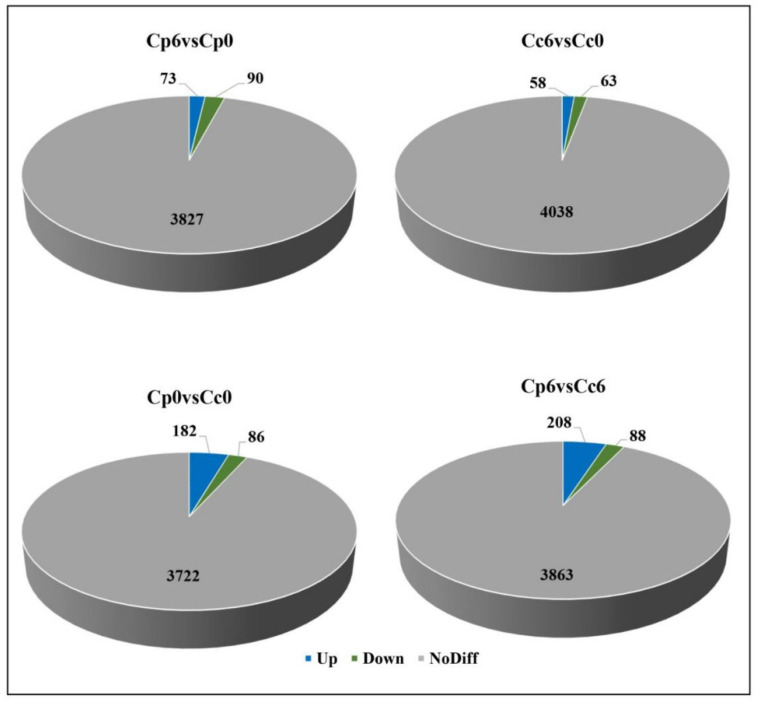
The overall number of differentially accumulated metabolites in the in the cold-treated *C. pubescens* and *C. chinense* pepper seedlings after 6 h of cold stress.

Leaf extracts from *C. pubescens* and *C. chinense* plants were analyzed using a liquid chromatograph-mass spectrometer to determine metabolic profiles. A total of 4159 metabolites were identified and quantified, including a wide range of primary and secondary metabolites, such as carbohydrates, organic acids, amino acids, flavonoids, lipids, terpenoids, and alkaloids. Under cold stress conditions, 121 metabolites (64 upregulated and 57 downregulated) were significantly altered in *C. pubescens*, and 126 metabolites (43 upregulated and 83 downregulated) were significantly altered in *C. chinense* plants after 6 h of cold stress (Figure 8). Notably, cold stress significantly increased some of the metabolites, such as a 2.8-fold increase in sucrose, 3.3-fold increase in UDP-glucose, 3.02-fold increase in fructose6phosphate, 2.4-fold increase in oxaloacetate, and 2.6-fold increase in asparagine, whereas some metabolites only increased in *C. pubescens* leaves, such as a 3.6-fold increase in maltose, 2.5-fold increase in ascorbate, 2.8-fold increase in gluconate, 3.4-fold increase in mannitol, 2.5-fold increase in proline, and 2.3-fold increase in arginine (Supplementary File S4).

### 2.9. KEGG Pathway Mapping of DAMs

**Figure 9:**
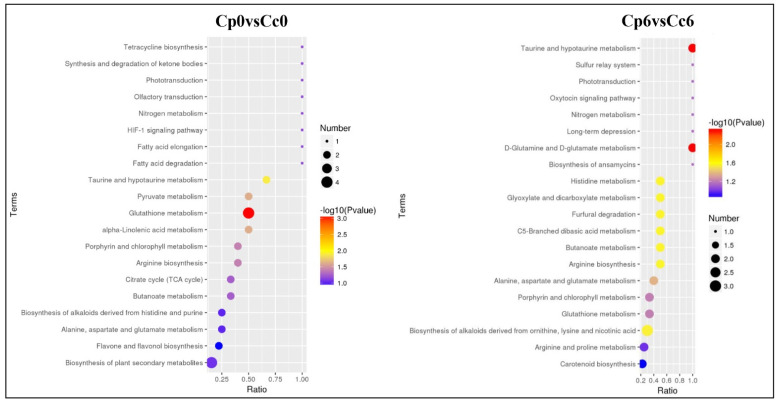
KEGG enrichment analysis of DAMs in the cold-treated *C. pubescens* and *C. chinense* pepper seedlings. The ordinate indicates the pathway name, and the abscissa is the enrichment factor. The size of the circle indicates the number of genes enriched in the KEGG pathway, and the color of the circle represents the *p*-value.

We used KEGG pathway analysis to determine which cellular pathways may be enriched for differentially expressed genes in different samples. These metabolic changes were primarily associated with carbohydrate and amino acid metabolism, the TCA cycles, and phenylpropanoid metabolism, suggesting a major role of these metabolic pathways in the signaling and response to cold stress (Figure 9, Supplementary File S5).

### 2.10. Conjoint Analysis of DEGs and DAMs Involved in Key Biological Pathways

**Figure 10:**
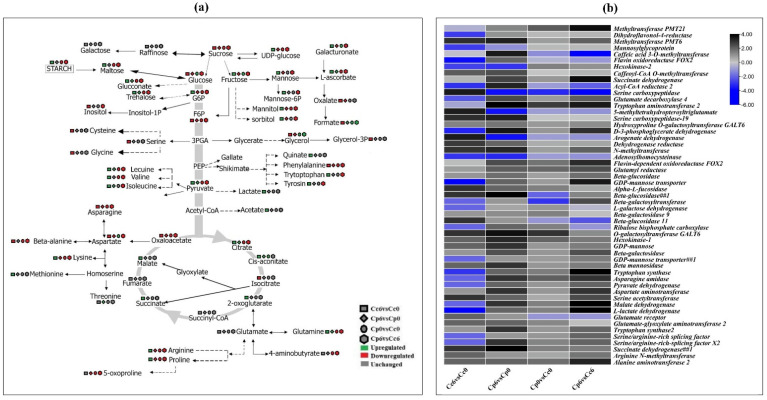
Overview of primary and secondary metabolic pathways during cold stress. (**a**) Changes in the metabolites associated with the pathways after cold stress in *C. pubescens* and *C. chinense* seedlings. (**b**) Expression patterns of the genes involved in metabolic pathways after cold stress in *C. pubescens* and *C. chinense* seedlings.

Sugars and amino acids are important regulatory factors in plant stress responses. Surprisingly, both metabolites and gene expression were engaged in amino acid and carbohydrate metabolism under cold stress. The amino acid and carbohydrate biosynthesis pathways are depicted schematically (Figure 10a). The majority of sugar and amino acid concentrations increased significantly in *C. pubescens* as the stress period was prolonged, whereas decreased contents were observed in *C. chinense*, suggesting that sugar and amino acid aggregation is critical in response to cold stress. Figure 10b depicts the expression patterns of the primary and secondary genes involved in metabolism. Cold stress resulted in a significant accumulation of primary and secondary metabolites. Most of the genes that are associated with the biosynthesis of these metabolites were upregulated, while some of the genes were downregulated. Cold stress increased the expression of genes encoding *GDP-mannose*, *aspartate aminotransmitase*, *galactosyltransferase*, *beta-glucosidase*, and *ribulose bisphosphate* (all of which are involved in sugar metabolism). Other amino acids (such as proline, alanine, quinate, tryptophan, and isoleucine) were found to be more abundant, and the expression levels of genes involved in their biosynthesis processes (*hydoxyproline O-galactosyltransferase*, *aspartate aminotransferase* (catalyzes the conversion of aspartate and alpha-ketoglutarate to oxaloacetate), *L-lactate dehydrogenase*, *succinate dehydrogenase*, *serine carboxypeptidase*, and *alanine aminotransferase 2*) were upregulated in *C. pubescens* plants under cold stress. On the other hand, arginine N-methyltransferase (it catalyzes the transfer of methyl groups from S-adenosyl-L-methionine to arginine residues) was downregulated in *C. chinense* but was expressed higher in *C. pubescens* under cold stress. This could explain the lower arginine content of *C. chinense* leaves compared to *C. pubescens*. Furthermore, in *C. pubescens* leaves, genes encoding secondary metabolism (caffeic acid 3-O-methyltransferase, flavin-dependent oxidoreductase, and caffeoyl-CoA O-methyltransferase) were upregulated in *C. pubescens* and were linked to higher levels of secondary metabolites under cold stress (Supplementary File S1).

### 2.11. Validation of Transcriptomic Data

**Figure 11:**
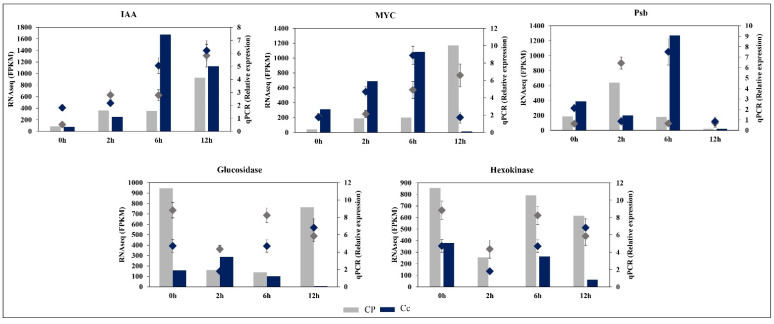
The expression profiles of significantly differentially expressed genes were measured by RNA-seq and qRT PCR. The histogram data represent the RNA-seq data (left Y-axis) and the marker represents the qRT-PCR data (right Y-axis).

We selected five genes related to secondary metabolism, photosynthesis, and hormone signal transduction for qRT-PCR verification. The results showed that the expression of each gene obtained by qRT-PCR analysis was relative to the FPKM value obtained by the RNA-seq data, which confirms the reliability of the gene expression data measured by RNA-seq (Figure 11). 

## 3. Discussion

Low temperatures often affect plant growth and crop productivity, which causes significant crop losses [21]. The plant response to low-temperature stress is a multi-factor mechanism involving diverse physiological, genomic, and metabolic regulatory networks. Pepper is a tropical crop and is susceptible to cold temperatures; however, the cold tolerance mechanism of the pepper plant still needs to be established. 

Our study identified the pepper response to cold stress in an integrated manner that combined transcriptomic and metabolomics approaches of cold-tolerant and cold-sensitive pepper species. When subjected to environmental stress, plants accumulate more osmotic adaptive compounds, such as soluble sugars, proline, inorganic ions, and betaine [22]. MDA is a common byproduct of lipid peroxidation that can aggravate membrane damage and provides a sensitive oxidative injury diagnostic index [23]. In our study, the MDA concentrations in *C. chinense* increased rapidly under cold stress compared to *C. pubescens*, indicating severe membrane phospholipid peroxidation in *C. chinense* plants. Cold stress stimulates the development of ROS, and unsaturated fatty acid peroxidation changes the composition of the membrane, which is impaired as a result of cold stress. 

Cold acclimation, which requires various physiological, genetic, and biochemical modifications, allows plants to acclimate to low temperatures. *Low-temperature-induced* (LTI), *early dehydration-inducible* (ERD), and *responsive to desiccation* (RD) genes are among the cold-responsive genes. Some of these genes code for key enzymes involved in osmolyte biosynthesis, which improves cold stress tolerance. To achieve cold stress tolerance, cryoprotective proteins, soluble sugars, and proline are accumulated to stabilize the cellular osmotic potential [24]. Our studies also demonstrated five significantly upregulated expressions of dehydration-responsive elements in cold-tolerant species, which correlate with the accumulation of osmolytes (sucrose, MDA, and proline) during cold stress. With the production of ROS, SOD activity also increased under cold stress. ROS have a signaling pathway that uses redox-sensitive transcription factors, such as the MAPK signaling cascade, to trigger defense-related gene expression [25]. More than 90% of the MAPK signaling genes were induced at low temperatures in *Brachypodium distachyon* [26]. According to the annotation of the KEGG pathways, a total of 13 DEGs were involved in the MAPK-plant signaling cascade, and nearly all DEGs were enriched by cold stress (Supplementary File S1).

Many studies showed that TF transcriptional networks are associated with plant tolerance to abiotic stresses [27,28]. Following previous reports [29,30], our transcriptomic results showed that the majority of differential TFs belonged to the family *bHLH*, *NAC*, *ERF*, *MYB*, and *WRKY* TFs, suggesting a variety and sophistication of regulatory approaches to the cold stress response in pepper plants. With 34 upregulated and 24 downregulated DEGs in the Cp12vsCc12 comparison, the *ERF* subfamily was the leading cold-stress-responsive TF family in cold-tolerant species. A previous study showed an essential role of *CBF*/*DREB* from an *ERF* superfamily in cold stress tolerance [31,32,33]. There is increasing evidence of the *WRKY* family’s involvement in plant stress reactions [33,34]. The upregulation of 14 *WRKY* family members in *C. pubescens* is of particular interest. This TF family’s overrepresentation may also point to their significance in increasing cold response tolerance in *C. pubescens*. The plant-specific *NAC* TF family plays a role in regulating multiple pathways, including plant growth, hormonal signals, and stress responses. In *Arabidopsis*, *RD26*, which codes for a *NAC* TF, has been shown to significantly improve stress response resistance [35]. We discovered eight *NAC* TF genes, and almost all of them showed upregulated expression in *C. pubescens* compared to *C. chinense*. These findings emphasize the significance of TFs in improving *C. pubescens* cold tolerance. Moreover, some members of the *bHLH* and *MYB* TF families were downregulated. *bHLH* and *MYB* TFs were also found to be associated with abiotic stress responses [35,36] and to interact with one another to regulate transcription [37]. As a result, we concluded that these TFs play a parallel role in response to abiotic stress conditions. 

Metabolites are the final products of cellular activities, and they represent the environmental effects on plants. In our study, several sugar components, including glucose, fructose, inositol, galactinol, and raffinoid, were upregulated. Trihalose and sucrose were not (Supplementary File S4). Meanwhile, many sugars not previously described for cold responses, such as tagatose and mannose, were observed. These findings may explain the fact that peppers still require sugars to maintain growth under short-term stress conditions. Moreover, major variations are accumulated in certain amino acids, such as leucine, valine, and tyrosine, in *C. pubescens*, whereas glycerol and serine were accumulated to a higher ratio in *C. chinense* plants (Figure 10a, Supplementary File S4). Previous research has shown that many stress-responsive plant metabolites are synthesized via the amino acid metabolic pathway, implying that amino acid metabolism plays a significant role in plant stress responses [38]. Flavonoids also play an essential role in alleviating the heat stress response by eliminating ROS produced by heat stress [39]. An enriched accumulation of flavonoids suggests their role not only in heat stress but also to reduce cold stress damage. These findings indicate that advances in genetic and metabolic pathways are highly effective in reducing cold stress damage. In the meantime, multiple genes involved in the synthesis of amino acids, sugars, and flavonoid metabolism and the aggregation of specific cold-responsive metabolites shed light on the cold stress response that can help elucidate the molecular mechanism of cold stress tolerance. Moreover, the general alignment of the transcriptome and qPCR findings, with the exception of glucosidase and hexokinase at the 12 h time point, suggests a thorough investigation of all genes of interest in future studies. 

## 4. Materials and Methods

### 4.1. Plant Material and Cold Treatment

Pepper seeds of *C. pubescens* (Entry No.: HNUCP0001) and *C. chinense* (Entry No.: HNUCC0001) were collected from the pepper germplasm bank of Hainan University. Seeds were grown after sterilization, and seedlings were transferred to the growth chamber (26 °C and 16 h/8 h light) for cultivation. After the emergence of 6–8 leaves, healthy seedlings were transferred into an incubator (MLR-352-PC) for cold treatment. Healthy and even seedlings (24 seedlings for each species) were cold-treated at 4 °C, and samples were collected from individual seedlings at different points (0 h, 2 h, 6 h, and 12 h), frozen immediately in liquid nitrogen, and stored at −80 for further analysis.

### 4.2. Morphological and Physiological Index Measurements 

The leaves were sampled to measure physiological indexes after 0 h (control), 2 h, 6 h, and 12 h of cold treatment at 4 °C. All analyses were performed using three biological replicates. The accumulation of malondialdehyde (MDA) was measured using the TBA chroma measurement method (Solarbio, Beijing, China). The SOD and POD activities were determined based on the instructions of the marketing kit (Beijing Sun Bio, China). Fresh leaves were ground using liquid nitrogen, and extraction buffers were used for enzyme extraction. The lyophilized material was then extracted at 8000× *g* and centrifugally extracted for 10 min at 4 °C. A spectrophotometer was used to determine the absorbance at 560 nm and 470 nm for SOD and POD activity, respectively. The production of superoxides was determined by a spectroscopic method based on nitro blue sulfur trioxide (NBT) detection, as previously described by [40]. Photosynthesis pigments were estimated using acetic acid extraction (80% acetate ketone), and absorptions were measured at 470 nm, 646 nm, and 663 nm using a spectrophotometer (Beckman 640D, Brea, CA, USA). The charcoal method was used to analyze the soluble sugar contents. In brief, 0.2 g of plant material (leaves) was extracted for 60 min with 80% ethanol. The total amount of soluble sugar was analyzed by adding freshly prepared reagents to 0.1 mL of alcohol extract. After extracting the soluble sugar, the solid part was used to extract the starch with 30% perchloric acid for 60 min at 60 °C. The optical density of the starch solutions was measured at 625 nm using D-glucose as the standard (range 0–100 micrograms mL^−1^) [41].

### 4.3. RNA Extraction, cDNA Library Construction, and Sequencing

The NEB extraction kit was used to extract RNA from all samples according to the manufacturer’s protocol. A NanoPhotometer^®^ (IMPLEN, CA, USA) was employed to evaluate RNA purity. The sequencing library was constructed using an RNA Library Prep Kit for Illumina^®^ (NEB, USA) as directed by the manufacturer. A TruSeq PE Cluster Kit v3-cBot-HS (Illumina) was used to perform cBot cluster generation. The library was sequenced on the Illumina Novaseq platform after cluster creation, yielding a 150 bp paired-end read. The raw data of HNUCC0001 at 0 h and 12 h were obtained from our previous study [42]. Three biological replicates were used for each sample. 

### 4.4. Quality Control and Mapping Reads to the Reference Genome

In the first step, the raw reads were processed using an internal Perl script that was written to support fastq processing. By deleting adapter-containing reads, clean data (clean reads) were obtained. Only clean and high-quality data were used to carry out all downstream analyses. Clean reads were aligned to the pepper reference genome (https://www.ncbi.nlm.nih.gov/genome/?term=Capsicum+chinense) using HISAT2 (v.2.0.5) [43] (accessed on 1 August 2020).

### 4.5. Differential Gene Expression Quantification

Feature Counts v1.5.0-p3 was used to calculate the number of reads mapped to each gene. The number of reads mapped to each gene was calculated, and based on the fragments per kilobase per million (FPKM), transcript sequences were mapped for each gene. FPKM takes account of sequencing depth and gene length as factors in determining read counts and is now the most frequently used technique for measuring gene expression levels. A differential expression analysis was carried out using the DESeq2 R package (1.16.1) on the two groups (including the biological replicates of each group). DESeq2 provides statistical procedures for digital gene expression data analysis using a model based on the negative binomial distribution, which calculates the total difference between genes being expressed higher or lower in an experiment. Benjamini and Hochberg’s method [44] was used to adjust the *p*-value to monitor the false discovery rate. Genes found by DESeq2 with an adjusted *p*-value < 0.05 were considered differentially expressed genes (DEG).

### 4.6. Weighted Gene Co-Expression Network Analysis (WGCNA) 

Voom-transformed expression data from the RNA-seq experiments were used as the input, and a weighted gene network was generated by following the standard procedure of the WGCNA package. 

### 4.7. KEGG Enrichment Analysis

The KEGG database is a resource that researchers can use to understand how the various functions and utilities of biological systems, such as cells, organisms, and ecosystems, work at the molecular level, particularly when it comes to genome sequencing and other high-level large-scale molecular data sets. To analyze the statistical enrichment of differentially expressed genes in the KEGG pathway, we utilized the clusterProfiler R program. When the rich factor, *p*-value, and the number of genes in the pathway are significant, it indicates a higher degree of KEGG enrichment. 

### 4.8. Metabolic Profiling Analysis 

Samples from *C. pubescens* and *C. chinense* were collected after 0 h and 6 h of cold stress for metabolic profiling. Next, 100 mg of dry tissue was powdered in liquid nitrogen, and the resulting powder was then suspended in 80% methanol and 0.1% formic acid using a vortex. The tissue samples were incubated on ice for 5 min, then subjected to centrifugation at 15,000 RPM at 4 °C for 5 min. To prepare the diluted supernatant, the suspension was diluted to a final concentration of 60% with LC-MS-grade water. The material was then transferred to a 0.22 μm filtered Eppendorf tube and subjected to centrifugation at 15,000× *g* at 4 °C for 10 min. In the last step, the filtrate was injected into the LC-MS/MS apparatus for analysis. A higher resolution LC-MS/MS coupled with an Orbitrap Q Exactive series mass spectrometer (Thermo Fisher, Newton Drive, Carlsbad, CA, USA) and a Vanquish UHPLC system (Thermo Fisher) was used.

### 4.9. Statistical Analysis, Database Search, and Data Analysis for Metabolites

To conduct the statistical analysis, the Social Science Statistical Package (SPSS) software (IBM SPSS Statistics, version 21.0, Armonk, NY, USA) was used via an analysis of variance (ANOVA). The third edition of Compound Discoverer (CD3.0, Thermo Fisher) was used to handle the raw data files generated by UHPLC-MS/MS. For the sake of this analysis, we specified the following parameters: signal-to-noise ratio, 3; retention time tolerance, 0.2 min; real mass tolerance, 5 ppm; signal strength tolerance, 30%. The peak intensity was normalized to the entire spectral intensity, at which point it became constant. The normalized data were utilized to forecast molecular formulae based on fragment ions, extra ions, and molecular ion peaks. Next, the mzCloud and ChemSpider databases were used to find peaks and then compare them to the spectra of compounds to obtain accurate qualitative and relative quantitative results. The identified metabolites were annotated using the KEGG database. The principal component analysis (PCA) was employed on the metaX program, which is a versatile and extensive software program that allows for multivariate analysis of all of the metabolites that have been discovered. Metabolites of variable importance in the prediction (VIP) > 1 with *p*-values < 0.05 and fold changes ≥ 2 or FC ≤ 0.5 were considered differential metabolites. These data were utilized to perform functional analyses for these metabolites and metabolic pathways. 

### 4.10. Quantitative Real-Time RT-PCR Analysis

Total RNA was collected from leaf tissue, and reverse transcription was performed using HiScript III (+gDNA wiper) (Vazyme, Nanjing, China). The SYBR qPCR Mix was used for qPCR analysis. The expression of each sample was calculated using three biological and three technical replicates. The primers used in this study are listed in Table S2.

## 5. Conclusions

The current study uncovered complex cold stress responses by transcriptomic and metabolomic reprogramming. Furthermore, we elucidated the metabolic pathways involved in pepper tolerance under cold stress. We successfully identified several genes and metabolites associated with critical biological pathways in the cold stress reaction based on transcriptomic and metabolomic datasets. To our knowledge, this is the first study to examine *C. pubescens* and *C. chinense* under cold stress, and it reveals a diverse regulatory network and multiple signaling pathways working together. Together, these results explain the comprehensive gene regulatory and metabolic networks involved in pepper cold tolerance.

## Data Availability

Supporting data can be found at https://www.ncbi.nlm.nih.gov/ under the project number PRJNA822667 (accessible on 1 May 2022).

## Supplementary Materials

The following supporting information can be downloaded at: https://www.mdpi.com/article/10.3390/ijms23126683/s1.
