# Supplementary material for: Integrated Transcriptomic and Metabolomic Analyses of Cold-Tolerant and Cold-Sensitive Pepper Species Reveal Key Genes and Essential Metabolic Pathways Involved in Response to Cold Stress
Source: Int J Mol Sci. 2022 Jun 15;23(12):6683. doi: 10.3390/ijms23126683 (PMC9224482; doi:10.3390/ijms23126683)
Supplement: Supplementary file 1 [file ijms-23-06683-s001.zip › Figure S1.pdf]

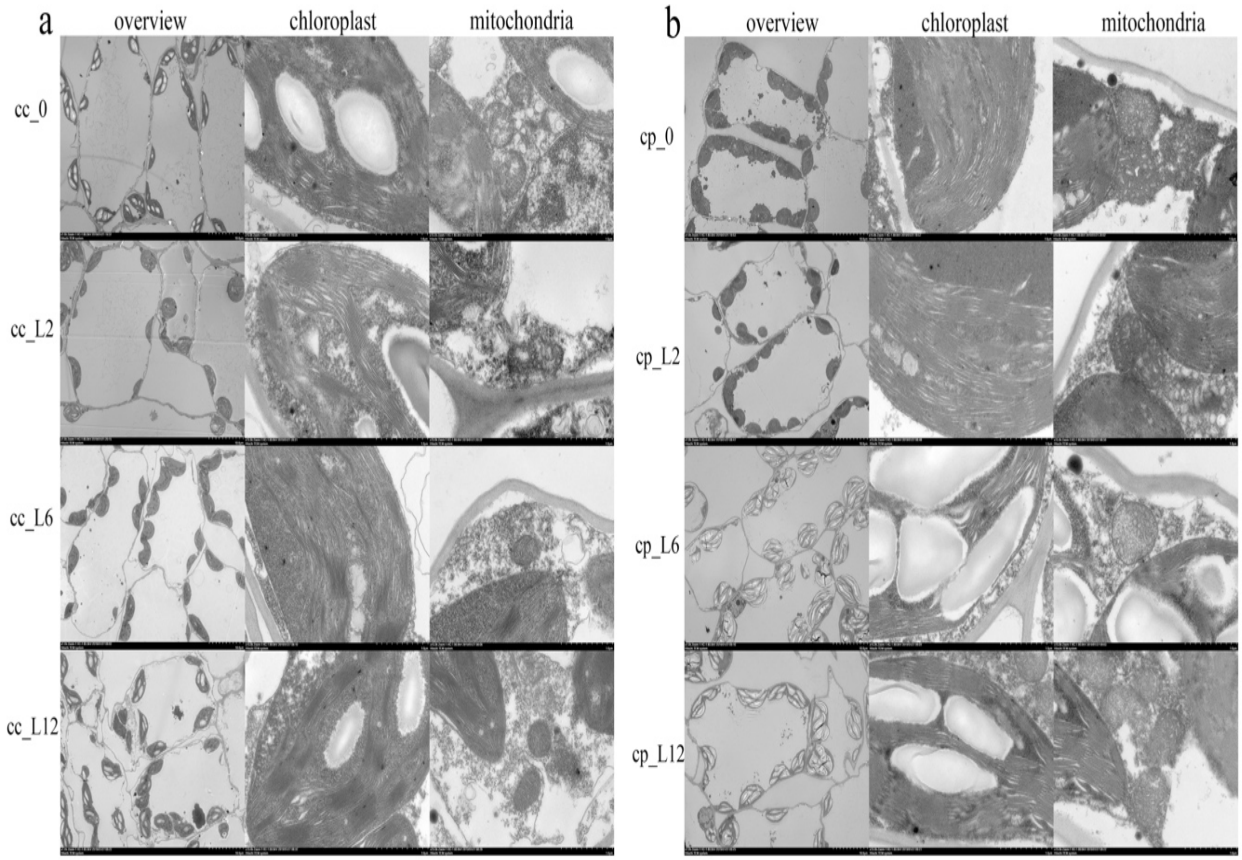

Figure S1. Transmission electron microscope images of *Cp* and *Cc* pepper leaves under cold stress at different time points. Scale bars = 10 μm. Sections were examined with a Phillips Morgagni 268 transmission electron microscope at an accelerating voltage of 80 kV. Digital images were recorded with a MegaViewIII digital camera operated with iTEM software.
