# Supplementary material for: Integrated Transcriptomic and Metabolomic Analyses of Cold-Tolerant and Cold-Sensitive Pepper Species Reveal Key Genes and Essential Metabolic Pathways Involved in Response to Cold Stress
Source: Int J Mol Sci. 2022 Jun 15;23(12):6683. doi: 10.3390/ijms23126683 (PMC9224482; doi:10.3390/ijms23126683)
Supplement: Supplementary file 1 [file ijms-23-06683-s001.zip › Table S1.pdf]

Table S1. Summary of sequencing data for different samples

Sample	Raw_reads	Clean_reads	Clean_bases	Error_rate	Q20	Q30	GC_pct
**Cc0-1**	45048498	44170816	6.63G	0.03	95.69	89.24	42.05
**Cc0-2**	48891838	47642794	7.15G	0.03	95.97	89.78	42.31
**Cc0-3**	52082416	50807534	7.62G	0.03	95.84	89.55	42.37
**Cc2-1**	57697044	56877384	8.53G	0.03	94.65	87.14	41.98
**Cc2-2**	43517342	42798150	6.42G	0.03	95.23	88.43	42.04
**Cc2-3**	41447528	40628878	6.09G	0.03	95.58	89.08	42.17
**Cc6-1**	63743738	61933538	9.29G	0.03	95.73	89.19	42.07
**Cc6-2**	53216086	51654824	7.75G	0.03	95.65	89.12	42.05
**Cc6-3**	42768604	41326264	6.2G	0.03	95.89	89.61	41.71
**Cc12-1**	49641942	47185700	7.08G	0.03	97.16	92.27	41.62
**Cc12-2**	46722346	45644374	6.85G	0.03	95.35	88.42	42.02
**Cc12-3**	53160708	51814502	7.77G	0.03	95.48	88.88	42.1
**Cp0-1**	66453898	65208758	9.78G	0.03	95.18	88.33	42.27
**Cp0-2**	60439190	59653034	8.95G	0.03	95.25	88.47	42.24
**Cp0-3**	59372212	58150980	8.72G	0.03	95.92	89.68	42.15
**Cp2-1**	41070852	40266368	6.04G	0.03	95.56	89.03	42.39
**Cp2-2**	46205440	44465114	6.67G	0.03	97.7	93.56	42.36
**Cp2-3**	46450528	44976660	6.75G	0.03	95.87	89.78	41.79
**Cp6-1**	56955370	55749512	8.36G	0.03	95.95	89.69	41.94
**Cp6-2**	49412636	46900584	7.04G	0.03	97.65	93.49	42.17
**Cp6-3**	45795510	44848932	6.73G	0.03	95.39	88.68	42.23
**Cp12-1**	61485338	59113302	8.87G	0.03	95.7	89.27	41.78
**Cp12-2**	49182128	47878336	7.18G	0.03	95.4	88.74	41.92
**Cp12-3**	43444776	42936310	6.44G	0.03	95.82	89.49	41.92
