# Supplementary material for: Integrated Transcriptomic and Metabolomic Analyses of Cold-Tolerant and Cold-Sensitive Pepper Species Reveal Key Genes and Essential Metabolic Pathways Involved in Response to Cold Stress
Source: Int J Mol Sci. 2022 Jun 15;23(12):6683. doi: 10.3390/ijms23126683 (PMC9224482; doi:10.3390/ijms23126683)
Supplement: Supplementary file 1 [file ijms-23-06683-s001.zip › Table S2.pdf]

Table S2. List of primers used for qRT-PCR analysis.

**IAA**	FW	CAGAGGAGTCTGGAGTTCGC
	RV	ACGTTCTCCGGCAGTTGATT
**Hexokinase**	FW	TAGCCAACAAACAGAACCCC
	RV	GGCTGTGGCATTGACAGTTT
**Psb**	FW	AACTCGTGTGCTTGGGAGTC
	RV	GAAGCGACCCCATAGGCTTT
**MYC**	FW	ACAGCTGATCAATCTGGTGTGA
	RV	AATTCCGTCTCGCGATTGGT
**Glucosidase**	FW	TGTTCCTCGGAATGTGGAGC
	RV	CTTCCGCAACCGATCCTCTT
